# Assessing quality of life using WHOQOL-BREF: a cross-sectional study on the association between quality of life and neighborhood environmental satisfaction, and the mediating effect of health-related behaviors

**DOI:** 10.1186/s12889-018-5942-3

**Published:** 2018-09-12

**Authors:** Fiona Y. Wong, Lin Yang, John W. M. Yuen, Katherine K. P. Chang, Frances K. Y. Wong

**Affiliations:** 1 School of Optometry, The Hong Kong Polytechnic University, Hung Hom, Kowloon, Hong Kong; 2 School of Nursing, The Hong Kong Polytechnic University, Hung Hom, Kowloon, Hong Kong

**Keywords:** Quality of life, Psychological health, Neighborhood environment, Smoking, WHOQOL-BREF

## Abstract

**Background:**

Quality of life (QOL) is an important component in assessing people’s health. Environmental quality can influence people’s QOL in the physical health, psychological, social relationships and environment domains. QOL in the four domains, overall QOL and general heath of residents living in the Kowloon Peninsula of Hong Kong were assessed. The association between satisfaction with the neighborhood environment and QOL, and health-related behaviors which mediated the effect were investigated.

**Methods:**

A sample of 317 residents completed a questionnaire which comprised the WHOQOL-BREF (Hong Kong version) to assess QOL, the International Physical Activity Questionnaire (IPAQ) to study physical activities, and questions on satisfaction with the neighborhood environment, health-related behaviors and socio-demographics. One-way ANOVA and linear regression were used to study the associations between environmental satisfaction and QOL in the four domains, overall QOL and general health, followed by assessing the relationships between environmental satisfaction and the potential health-related behavior mediators with regression tests. Mediation analysis was conducted using multiple linear regressions to study the effects of environmental satisfaction on QOL in the four domains, overall QOL and general health, as well as the potential mediating roles played by various health-related behaviors. A *P*-value of < 0.05 was considered as statistically significant.

**Results:**

The residents had a relatively higher physical health mean score of 70.83 ± 12.69, and a lower environmental mean score of 61.98 ± 13.76. Moderate satisfaction with the neighborhood environment had a significant relationship with QOL in the psychological domain (β = 0.170, *P* = 0.006), however, this effect was partially mediated by the non-smoking behavior of the residents (β = 0.143, *P* = 0.022).

**Conclusions:**

Our residents had lower QOL in the physical health and psychological domains but similar QOL in the social relationships and environmental domains compared to other countries. Only QOL in the psychological domain could be predicted by the satisfaction with the neighborhood environment, and non-smoking status was a partial mediator of the effect of moderate environmental satisfaction on QOL in the psychological domain. Refrain from smoking seems to be able to lower the influence of neighborhood environment on people’s QOL in the psychological domain to a certain extent.

## Background

Quality of life (QOL) is an important component in assessing people’s health. It commonly focuses on physical and mental health and functional performance of individuals, however, QOL can be measured in a broad range. The World Health Organization Quality of Life: Brief Version (WHOQOL-BREF) assesses QOL in four domains including physical health, psychological, social relationships and environment [1]. QOL refers to individuals’ perception of their position in life in the context of the culture and value systems in which they live and in relation to their goals, expectations, standards and concerns. The QOL in the four domains can be affected by different factors like age, sex, rural and urban areas [2, 3], and health and disease status [4].

Concerns for environmental and greening impact on QOL and general health are rising these days. The quality of both the physical and built environment can influence people’s perception of general health, well-being and QOL, especially to the youth and elderly [5–7]. Air pollutants and toxins, noise and dirtiness have a negative impact on people’s QOL in the physical and psychological domains [8]. Pollution-related diseases, such as respiratory infections, asthma and cardio-pulmonary diseases, could lower people’s QOL [9]. Long-term noise exposure not only can cause hearing impairment, but also hypertension, ischemic heart disease, annoyance, sleep disturbance, depression and anxiety [10]. Features of the environment such as accessibility to green space and availability of safe parks, have been found positively associated with mental health and negatively related to stress level [8, 11].

Besides environmental factors, QOL can also be affected by health-related behaviors. A study observed that participating in physical activities was associated with better QOL in both the physical and environmental domains [12], however, another study focusing on people with major depressive disorder found that physical activity could only improve QOL in the physical domain [13]. Walking for leisure was also positively associated with QOL in social relationships and environmental domains among men; however, it was positively associated with physical, psychological and environmental domains among women [14]. In addition to physical activities, people who practiced health behaviors like refrain from smoking and following a healthy diet were usually more psychologically healthy with less depressive symptoms [15, 16].

Social economic status (SES) including income, education and occupation could have a certain effect on the living environment of an individual. Previous studies found that those who had higher income and education level tended to live in less polluted neighborhood with more access to green and open spaces [10, 17]. The more healthy and green environment usually brings higher QOL especially in the psychological and environmental aspects [5, 7].

Hong Kong is a highly dense city with over 7 million populations, located at the southern coast of China with an area of approximately 1100km^2^ [18]. Although around 50% are woodland and shrub land, most of them are inaccessible. Open space is a luxury rather than a basic necessity. Only 2.3% of the lands are for parks, stadiums, playgrounds and recreational facilities. Air pollution and excessive noise are also long-lasting issues in Hong Kong because of crowdedness and heavy traffic. A previous study reported that environmental quality was one of the important infrastructures contributed to QOL of the university students in Hong Kong, however, environment and greening were the least satisfactory ones [19]. Since greenery and open spaces are limited in Hong Kong, we aimed to study the effect of satisfaction with the neighborhood environment on residents’ QOL, and the potential of encouraging health-related behaviors to improve QOL.

The objectives of this study were (i) to assess the QOL in the four domains, including physical, psychological, social relationships and environmental, and overall QOL and general heath, of residents living in the Kowloon Peninsula of Hong Kong, (ii) to study the effect of satisfaction with the neighborhood environment on QOL, and (iii) to identify health-related behaviors which mediated the effect of satisfaction with the neighborhood environment on QOL.

## Methods

### Target population

Hong Kong consists of three main territories including Hong Kong Island, Kowloon Peninsula and New Territories. There are 127 district council constituency areas in the Kowloon Peninsula. Nine district council constituency areas near Hung Hom and Tsim Sha Tsui located in the southwest of Kowloon Peninsula were selected as the target study sites. These areas cover mixed-use commercial and residential districts, old urban and more affluent districts with private and public housing, and people with low and high SES [20]. According to the 2011 Hong Kong Population Census, there were 158,103 people living in these areas [20]. Among this population, 88.0% were Chinese and 53.8% were female. Age distribution was comparable to the whole population in Hong Kong. Residents aged 18 years or above and had been living in the nine areas for at least 90 days were the target population. Those who were cognitively impaired or unable to communicate effectively in Cantonese, Mandarin and English were excluded. Assuming that 50% of the population was satisfied with the environment, a sample size of 266 would provide a precision of 6% from the true values at 95% confidence level.

### Instrument

The validated WHOQOL-BREF (Hong Kong version) was used to measure QOL [1, 21]. It consists of 24 items to assess perception of quality of life in four domains, including physical health, psychological, social relationships and environment, and two items on overall QOL and general health. The domain scores were transformed into a linear scale between 0 and 100 following the scoring guidelines [1]. A higher score indicated a better QOL. Physical activities were assessed by the International Physical Activity Questionnaire short form (IPAQ-SF) which is adequately reliable and valid in a Chinese population [22]. Walking, moderate-intensity and vigorous-intensity activities were assessed. The total MET-minutes/week was calculated for each participant. Metabolic Equivalent of Task (MET) is the energy cost of physical activities [23]. Physical activity levels were categorized as low, moderate and high, based on the criteria listed on the IPAQ guidelines [22]. People have to answer all questions on number of days in a week and daily time performing walking, moderate and vigorous activities in order to calculate their total MET and identify their physical activity levels. Those who were unable or refused to answer all these questions were removed from the analysis according to the IPAQ guidelines [22]. Smoking, alcohol drinking, and vegetable and fruit intakes were also assessed. For practicing low fat, low salt and low sugar diets, respondents could choose “never (1)”, “seldom (2)”, “sometimes (3)” and “always (4)”. Seven questions on satisfaction with the neighborhood environment with a five-point Likert scale (1: very unsatisfied; 5: very satisfied) were used to evaluate air quality, ventilation, drinking water quality, noise condition, lighting condition, environmental hygiene, and environmental protection and recycling. Six questions on satisfaction with open spaces including greening, parks and gardens, recreation and sports facilities, promenade, rest areas and pedestalization were also assessed using the same Likert scale. Socio-demographic profiles of the residents were also assessed.

Test-retest reliability of the questionnaire had been tested with 64 subjects before implementation of the main study. All question items showed a Cohen’s kappa value ranged from 0.50–1.00 or an intraclass correlation coefficient value ranged from 0.55–1.00, which indicated that the reliability of the questionnaire was fair to excellent.

### Procedures

This was a cross-sectional study using a convenience sampling method to recruit potential participants. Trained interviewers approached people at parks, resting areas and outside food markets and shopping centers in the nine district areas from 9 am to 7 pm. In addition to weekdays, the questionnaire interviews were also conducted on weekends to reach residents who spent most of their time in other districts on weekdays. After confirming the eligibility of participation, the purpose of the questionnaire survey was explained. If the potential participants were in a group, only one family member from each household was allowed to answer the questionnaire. A verbal consent was obtained from each of the participants. The answered questionnaire and the signing for receiving the token of appreciation were also the implied consent to participate in the study. A face-to-face interview was conducted on the recruitment site. A HKD 50 (approximately USD 6.45) supermarket shopping voucher was given as a token of appreciation to those who completed the questionnaire.

### Statistical analysis

Data analysis was performed using SPSS version 21. Descriptive statistics were reported by mean ± standard deviation or proportion, as appropriate. Satisfaction levels with physical environment and open spaces across different districts were compared using chi-square tests. Relationships between QOL in the four domains, overall QOL and general health were assessed using Spearman’s correlations.

**Fig. 1:**
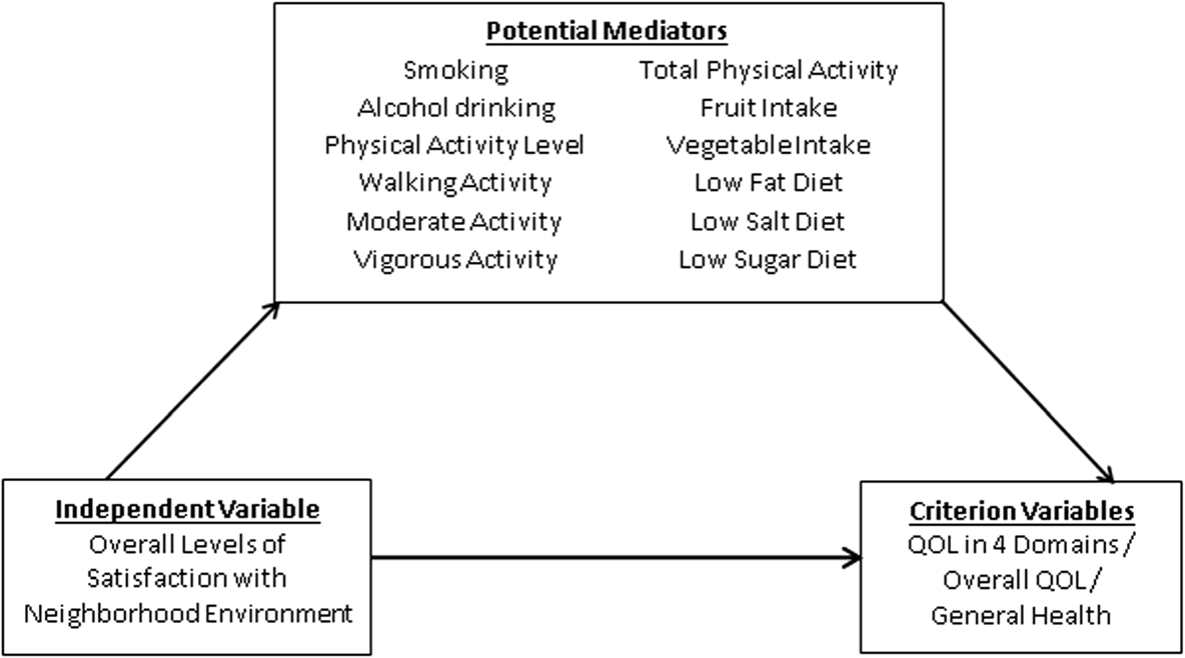
Hypothetical mediated model

Mediation analysis was conducted to study the effects of overall levels of satisfaction with the neighborhood environment (independent variable) on QOL in the four domains, overall QOL and general health (criterion variables), as well as the potential mediating roles played by various health-related behaviors (Fig. 1). To obtain the overall levels of satisfaction with the neighborhood enevironment, the mean scores of those environmental and open spaces variables which showed significant differences across the nine districts were summed to calculate the range of the overall environmental satisfactory scores (1: very unsatisfied; 5: very satisfied). Scores in the lowest 1/3 were categorized as “low satisfaction”; the second 1/3 were categorized as “moderate satisfaction”, while the highest 1/3 were categorized as “high satisfaction”. To conduct the mediation analysis, the existence of relationships between the independent variable and criterion variables were assessed using one-way ANOVA and linear regression in the first place, followed by assessing the existence of relationships between the independent variable and the potential health-related behavior mediators with regression tests. Mediated models with the combined effects of overall levels of satisfaction with the neighborhood environment and each of the health-related behaviors in predicting QOL were evaluated using multiple linear regression tests. Regression coefficients were used to assess the effect of mediation and further confirmed by both the Aroian and Goodman tests. A *P*-value of < 0.05 was considered as statistically significant.

## Results

### Socio-demographics

A total of 317 residents were successfully interviewed during July – September 2015. The majority were Chinese (99%), 44% were male, 60% were married and the mean age was 45.12 years (SD = 17.28) (Table 1). Nearly 46% had been living in the nine district areas for more than 10 years and 46% were living in a self-owned private permanent housing. About 33% had attained a university level. Around 25% claimed that they had no income while 31% received an individual monthly income more than HKD 14800 (USD 1922), the median monthly income of the Hong Kong population in 2014 [24].

**Table 1:** Socio-demographic characteristics and health-related behaviors of the residents (*N* = 317)

	*n* (%)	Mean ± SD
Sex		
Male	139 (43.8%)	/
Female	178 (56.2%)	
Age (years)		
18–24	48 (15.1%)	45.12 ± 17.28
25–44	107 (33.8%)	
45–64	115 (36.3%)	
≥ 65	47 (14.8%)	
Ethnicity		
Chinese	313 (99.1%)	/
Non-Chinese	3 (0.9%)	
Missing	1	
Length of residence in the district (yrs)		
**≤** 5	95 (30.0%)	14.94 ± 13.09
6–10	75 (23.7%)	
11–20	64 (20.2%)	
≥ 21	83 (26.2%)	
Types of housing		
Self-owned private permanent housing	145 (45.9%)	/
Rental private permanent housing	34 (10.8%)	
Rental public housing	87 (27.5%)	
Others	50 (15.8%)	
Missing	1	
Education		
Primary school or below	61 (19.2%)	/
Secondary school	117 (36.9%)	
Diploma/Certificate	33 (10.4%)	
University degree	106 (33.4%)	
Individual monthly income (HKD)		
$0	78 (24.6%)	/
$10,500 or below	83 (26.2%)	
$10,501–14,800	59 (18.6%)	
$14,801–23,000	45 (14.2%)	
≥ $23,001	52 (16.4%)	
Marital status		
Never married	110 (34.7%)	/
Married	191 (60.3%)	
Widowed/Divorced/Separated	16 (5.0%)	
Current smoking status		
Never smoke	284 (89.6%)	/
Occasionally	10 (3.2%)	
Everyday^a^/Almost every day	23 (7.3%)	
Alcohol drinking		
Never	165 (52.1%)	/
1 or < 1/mth	94 (29.7%)	
2–4/mth	40 (12.6%)	
2–3/wk	10 (3.2%)	
≥ 4/wk.	8 (2.5%)	
Fruit intake		
Never	13 (4.1%)	/
< 1 serving/wk	173(54.6%)	
1 serving/day	89 (28.1%)	
≥ 2 servings/day	42 (13.2%)	
Vegetable intake		
Never	5 (1.6%)	
< 1 serving/day	127 (40.1%)	
1 serving/day	92 (29.0%)	/
2 servings/day	69 (21.8%)	
≥ 3 servings/day	24 (7.6%)	
Practicing low fat diet		
Never	48 (15.1%)	/
Seldom	55 (17.4%)	
Sometimes	122 (38.5%)	
Always	92 (29.0%)	
Practicing low salt diet		
Never	51 (16.1%)	/
Seldom	60 (18.95)	
Sometimes	114 (36.0%)	
Always	92 (29.0%)	
Practicing low sugar diet		
Never	46 (14.5%)	/
Seldom	66 (20.8%)	
Sometimes	104 (32.8%)	
Always	101 (31.9%)	
Physical Activity Level (*N* = 240)		
Low	32 (13.3%)	/
Moderate	151 (62.9%)	
High	57 (23.8%)	
Types of Activities (MET-minutes/week)		
Walking	/	1343.60 ± 1075.91
Moderate-intensity activity		361.98 ± 759.74
Vigorous-intensity activity		357.92 ± 739.23
Total physical activity		2142.94 ± 1661.32

^a^Every day: one stick of cigarette a day or 7 sticks of cigarettes a week for at least 6 months

### Health-related behaviors

A total of 10.5% of the residents smoked every day, almost every day or occasionally (Table 1). About 18.3% claimed that they consumed alcohol at least 2–4 times/month. Regarding fruit and vegetable intakes, 13.2% and 7.6% had achieved the recommended intake of at least 2 servings of fruits and 3 servings of vegetables a day, respectively. More than 60% claimed that they sometimes or always practiced low fat, low salt or low sugar eating. For physical activity, those who were unable or refused to estimate their number of days or daily time performing walking, moderate and/or vigorous activities were removed from the analysis. 240 out of 317 respondents completed the IPAQ assessment. Most of them were categorized as having moderate physical activity level (62.9%), and 13.3% were having low activity level or not performing physical activity.

### Satisfaction with environment and open spaces

The residents were most satisfied with water quality (55.4%) in their living districts, whereas they were most dissatisfied with air quality (29.7%), ventilation (27.1%) and noise level (25.9%). For open spaces, they were most satisfied with the promenade (36.9%) but another 22.7% were dissatisfied with the promenade. On the other hand, 25.2% and 22.1% were dissatisfied with the pedestrianization and greening in the open spaces, respectively. Crosstabulations of the living districts and satisfaction with physical environment and open spaces found that satisfaction levels with air quality (*P* = 0.029), noise condition (*P* = 0.041), parks and gardens (*P* = 0.015), recreation and sports facilities (*P* = 0.034) and promenade (*P* < 0.001) were significantly different across the nine districts (Table 2).

**Table 2:** Satisfaction with physical environment and open spaces across the nine districts using chi-square

					n (%)					
	District 1 *N* = 12	District 2 *N* = 28	District 3 *N* = 48	District 4 *N* = 40	District 5 *N* = 40	District 6 *N* = 49	District 7 *N* = 17	District 8 *N* = 53	District 9 *N* = 30	*P*
Air quality										
Very dissatisfied/Dissatisfied	6 (50.0%)	8 (28.6%)	22 (45.8%)	14 (35.0%)	13 (32.5%)	12 (24.5%)	3 (17.6%)	10 (18.9%)	6 (20.0%)	0.029
Fair	4 (33.3%)	10 (35.7%)	19 (39.6%)	12 (30.0%)	17 (42.5%)	24 (49.0%)	11 (64.7%)	18 (34.0%)	13 (43.3%)	
Very Satisfied/Satisfied	2 (16.7%)	10 (35.7%)	7 (14.6%)	14 (35.0%)	10 (25.0%)	13 (26.5%)	3 (17.6%)	25 (47.2%)	11 (36.7%)	
Noise condition										
Very dissatisfied/Dissatisfied	5 (41.7%)	9 (32.1%)	18 (37.5%)	6 (15.0%)	9 (22.5%)	20 (40.8%)	3 (17.6%)	6 (11.3%)	6 (20.0%)	0.041
Fair	4 (33.3%)	8 (28.6%)	20 (41.7%)	16 (40.0%)	20 (50.0%)	13 (26.5%)	8 (47.1%)	23 (43.4%)	13 (43.3%)	
Very Satisfied/Satisfied	3 (25.0%)	11 (39.3%)	10 (20.8%)	18 (45.0%)	11 (27.5%)	16 (32.7%)	6 (35.3%)	24 (45.3%)	11 (36.7%)	
Parks and Gardens										
Very dissatisfied/Dissatisfied	2 (16.7%)	3 (10.7%)	7 (14.6%)	10 (25.0%)	7 (17.5%)	14 (28.6%)	2 (11.8%)	5 (9.4%)	3 (10.0%)	0.015
Fair	5 (41.7%)	9 (32.1%)	31 (64.6%)	16 (40.0%)	17 (42.5%)	25 (51.0%)	11 (64.7%)	21 (39.6%)	15 (50.0%)	
Very Satisfied/Satisfied	5 (41.7%)	16 (57.1%)	10 (20.8%)	14 (35.0%)	16 (40.0%)	10 (20.4%)	4 (23.5%)	27 (50.9%)	12 (40.0%)	
Recreation & Sports Facilities										
Very dissatisfied/Dissatisfied	3 (25.0%)	2 (7.1%)	17 (35.4%)	11 (27.5%)	11 (27.5%)	10 (20.8%)	4 (23.5%)	7 (13.2%)	3 (10.0%)	0.034
Fair	6 (50.0%)	14 (50.0%)	23 (47.9%)	12 (30.0%)	16 (40.0%)	25 (52.1%)	11 (64.7%)	23 (43.4%)	15 (50.0%)	
Very Satisfied/Satisfied	3 (25.0%)	12 (42.9%)	8 (16.7%)	17 (42.5%)	13 (32.5%)	13 (27.1%)	2 (11.8%)	23 (43.4%)	12 (40.0%)	
Promenade										
Very dissatisfied/Dissatisfied	2 (16.7%)	3 (10.75)	12 (25.0%)	13 (32.5%)	3 (7.5%)	14 (28.6%)	6 (35.3%)	14 (26.4%)	5 (16.7%)	< 0.001
Fair	5 (41.7%)	8 (28.6%)	29 (60.4%)	10 (25.0%)	11 (27.5%)	24 (49.0%)	9 (52.9%)	15 (28.3%)	17 (56.7%)	
Very Satisfied/Satisfied	5 (41.7%)	17 (60.7%)	7 (14.6%)	17 (42.5%)	26 (65.0%)	11 (22.4%)	2 (11.8%)	24 (45.3%)	8 (26.7%)	
Overall Environmental Satisfactory Score, Mean ± SD	15.17 ± 3.54	16.96 ± 3.29	14.35 ± 3.04	15.40 ± 3.76	15.78 ± 2.83	14.94 ± 3.47	14.94 ± 1.64	16.72 ± 2.86	16.00 ± 2.69	
Environmental Satisfactory Level	Low	High	Low	Moderate	Moderate	Low	Low	High	Moderate	

### Quality of life

Table 3 shows the scores of QOL in the four domains, overall QOL and general health. Comparing the four domains of the residents, physical health domain was the highest with a mean score of 70.83 ± 12.69 while the environmental domain was the lowest with a mean score of 61.98 ± 13.76. Using one SD below the mean as the cut-off standards for low QOL [23], 18.9% of the respondents were considered to have poor QOL in the physical domain, followed by environmental domain (16.7%), psychological domain (14.5%) and social relationships domain (10.4%). The four domains, overall QOL and general health were significantly and positively interrelated with low to moderate relationships (r = 0.30–0.53, *P* < 0.01) (Table 3).

**Table 3:** Scores of the 4 QOL domains, overall QOL and general health and their spearman’s correlations (*N* = 317)

	Mean	SD	Number of participants with poor scores^a^, *n* (%)	General Health	Overall QOL	Environmental QOL	Social Relationships QOL	Psychological QOL	Physical QOL
				Spearman’s correlations (r)					
Domain 1 Physical QOL	70.83	12.69	60 (18.9%)	0.43**	0.32**	0.44**	0.34**	0.53**	1.00
Domain 2 Psychological QOL	65.43	12.61	46 (14.5%)	0.41**	0.38**	0.49**	0.51**	1.00	
Domain 3 Social Relationships QOL	63.96	14.61	33 (10.4%)	0.30**	0.31**	0.33**	1.00		
Domain 4 Environmental QOL	61.98	13.76	53 (16.7%)	0.32**	0.41**	1.00			
General QOL	62.07	15.98	14 (4.4%)	0.36**	1.00				
General health	60.41	18.50	31 (9.8%)	1.00					

^a^scores <1SD

**P < 0.01

### Mediation analysis

#### Overall levels of satisfaction with the environment

To calculate the overall environmental satisfactory scores, the mean scores of the physical environment and open spaces variables which showed significant differences across the nine districts including air quality, noise condition, parks and gardens, recreation and sports facilities, and promenade, were summed. The overall environmental satisfactory scores in the lowest 1/3 (14.35–15.22) were categorized as “low satisfaction”; the second 2/3 (15.23–16.09) were categorized as “moderate satisfaction”, while the highest 1/3 (16.10–16.96) were categorized as “high satisfaction” (Table 2).

#### Associations between the independent variable and the criterion variables

One-way ANOVA tests showed that the overall levels of satisfaction with the neighborhood environment only significantly predicted QOL in the psychological domain (*P* = 0.020) (Table 4). Further analysis using linear regression found that residents with moderate satisfaction with the neighborhood environment had significantly higher QOL in the psychological domain (β(95%CI) = 0.170 (1.30–7.72), *P* = 0.006). Associations between the overall levels of satisfaction with the environment and the other three QOL domains, overall QOL and general health were insignificant.

**Table 4:** Associations between overall levels of satisfaction with neighborhood environment and QOL

	Physical QOL			Psychological QOL			Social QOL			Environmental QOL			Overall QOL			General Health		
	Mean Square	F	P	Mean Square	F	P	Mean Square	F	P	Mean Square	F	P	Mean Square	F	P	Mean Square	F	P
Environment Satisfaction	202.52	1.26	0.285	621.53	3.98	0.020	218.73	1.03	0.360	502.83	2.68	0.070	168.67	0.66	0.518	870.15	2.57	0.078
	β (95% CI)		P	β (95% CI)		P	β (95% CI)		P	β (95% CI)		P	β (95% CI)		P	β (95% CI)		P
Environment Satisfaction^a^																		
Moderate	0.10 (− 0.65−5.86)		0.116	0.17 (1.30–7.72)		0.006	0.09 (− 1.09–6.41)		0.164	0.12(− 0.10–6.93)		0.057	0.03 (−3.03–5.19)		0.606	0.12 (− 0.22–9.23)		0.062
High	0.03 (− 2.66−4.44)		0.622	0.04 (−2.30–4.71)		0.499	0.06 (− 2.26–5.93)		0.378	0.12 (0.06–7.73)		0.046	−0.04 (− 6.09–2.87)		0.481	0.12 (− 0.04–10.27)		0.052

^a^The overall satisfactory levels of environment were converted to dummy variables

#### Associations between the independent variable and the potential mediators

Regression analyses showed that overall levels of satisfaction with the neighborhood environment were significantly related to smoking status, alcohol drinking, physical activity level, and walking activity and total physical activity measured in MET-minutes/week (*P* < 0.05) (Table 5). They were the potential mediators which might have an effect with the overall levels of satisfaction with the neighborhood environment in predicting QOL in the psychological domain. Fruit and vegetable intakes, practice of low fat, low salt and low sugar diet eating, and moderate and vigorous activities were not significantly related to the overall levels of satisfaction with the neighborhood environment.

**Table 5:** Associations between overall levels of satisfaction with neighborhood environment and health-related behaviors

	Environment Satisfaction		
	X^2^ ^a^		*P*
Smoking status	9.97		0.007
Alcohol drinking	13.19		0.010
Fruit intake	4.95		0.084
Vegetable intake	4.84		0.089
Low fat diet	3.76		0.709
Low salt diet	4.00		0.677
Low sugar diet	4.02		0.674
Physical activity level	12.04		0.017
	Mean Square ^b^	F	*P*
Walking activity	55,503,381	4.89	0.008
Moderate activity	794,945.73	1.38	0.253
Vigorous activity	1,149,574.02	2.12	0.122
Total physical activity	12,137,648.59	4.53	0.012

^a^Multinominal regression test

^b^Linear regression test

#### Associations between the potential mediators and the criterion variable

Each of the potential health-related behaviors identified was tested with the combined effect of overall levels of satisfaction with the neighborhood environment in predicting QOL in the psychological domain. Table 6 showed the results of each of the models.

**Table 6:** Effects of overall levels of satisfaction with neighborhood environment and health-related behaviors in predicting psychological QOL

	Psychological QOL			
	Multiple Linear Regression		Pearson’s Correlation	
	β	*P*	r	*P*
Model 1				
Environment Satisfactory levels*				
Moderate	0.143	0.022	0.153	0.003
High	0.036	0.560	−0.031	0.291
Smoking status*				
Non-smoking	0.157	0.005	0.178	0.001
Model 2				
Environment Satisfactory levels*				
Moderate	0.180	0.004	0.153	0.003
High	0.042	0.498	−0.031	0.291
Alcohol drinking*				
Never	0.070	0.575	0.021	0.356
Occasionally	0.024	0.849	−0.020	0.364
Model 3				
Environment Satisfactory levels*				
Moderate	0.133	0.071	0.135	0.019
High	0.006	0.937	−0.049	0.224
Physical activity levels*				
High	0.180	0.056	0.080	0.108
Moderate	0.143	0.128	0.012	0.427
Model 4				
Environment Satisfactory levels*				
Moderate	0.170	0.014	0.146	0.009
High	0.030	0.669	−0.030	0.315
Walking activity	0.105	0.090	0.095	0.061
Model 5				
Environment Satisfactory levels*				
Moderate	0.133	0.071	0.135	0.019
High	−0.003	0.964	− 0.049	0.224
Total physical activity	0.103	0.117	0.103	0.056

^*^The overall satisfactory levels of environment, smoking status, alcohol drinking, and physical activity levels were converted to dummy variables

Referring to the beta coefficients and Pearson’s correlations, only non-smoking was significantly correlated with QOL in the psychological domain (β = 0.157, *P* = 0.005; r = 0.178; *P* = 0.001) (Model 1). Non-smokers (64.03 ± 12.68) were found to have significantly higher QOL in the psychological domain than those who smoked occasionally (61.92 ± 9.22) and every day (61.74 ± 9.36). In the non-mediated model, moderate satisfaction with the neighborhood environment significantly predicted QOL in the psychological domain (β =0.170, *P* = 0.006) (Table 4), however, when mediated by non-smoking status, the beta weight was lowered to 0.143 (*P* = 0.022) but remained significant (Model 1). This indicated that the effect of moderate satisfaction with the neighborhood environment on QOL in the psychological domain was partially mediated by the non-smoking status of the residents.

Other health-related behaviors including alcohol drinking, physical activity levels, walking activity and total physical activity were not significantly related to QOL in the psychological domain (Models 2–5).

#### Quantifying the partial mediation effect

**Fig. 2:**
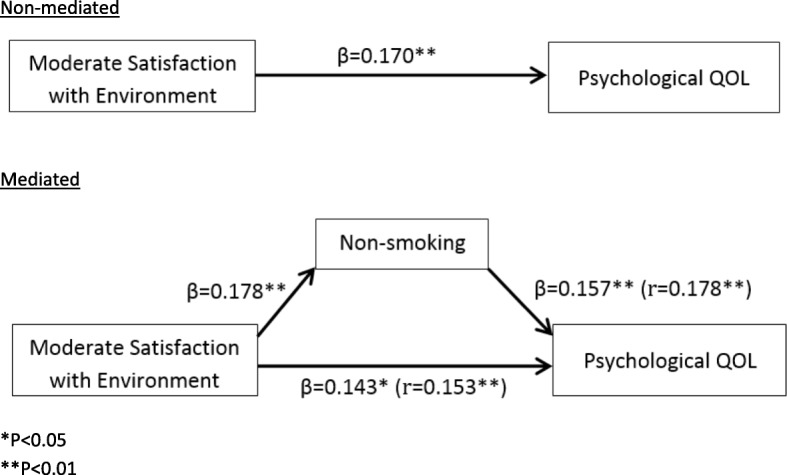
The non-mediated and mediated models with path coefficients. *P < 0.05, **P < 0.01

The statistical significance of the mediation effect was further confirmed by both the Aroian test (Z = 1.964, SE = 0.375, *P* = 0.049) and the Goodman test (Z = 2.088, SE = 0.353, *P* = 0.037). The beta coefficient of the effect of moderate satisfaction with the neighborhood environment on non-smoking status (β = 0.178, *P* = 0.004) (not shown), and the beta coefficient of the effect of non-smoking status on QOL in the psychological domain (β = 0.157, *P* = 0.005), gave a product of beta coefficient of 0.0279 (0.178 × 0.157) in the mediated path. The indirect effect of 0.0279 and the total direct effect of 0.170 gave a ratio of 0.1641, which indicated that 16.41% of the effect of moderate satisfaction with the neighborhood environment on QOL in the psychological domain was mediated through the non-smoking status (Fig. 2). Part of the relationship between moderate satisfaction with neighborhood environment and QOL in the psychological domain was due to the effect of non-smoking status on the psychological domain.

## Discussion

Among the four domains of QOL, the residents in this study had a relatively higher QOL in the physical health domain and a lower QOL in the environmental domain. An overall moderate satisfaction with the neighborhood environment was found significantly predicted QOL in the psychological domain, however, this effect was partially mediated by the non-smoking status of the residents.

This study covered both where the affluent and the relatively poor people were living. According to the census statistics, the demographic characteristics of our respondents were comparable to the whole population in Hong Kong in terms of sex and age [25, 26]. However, our respondents were more educated, mostly married and received lower monthly income.

Our residents showed lower QOL in the physical health and psychological domains compared with the mean scores reported by the 23 countries in the WHOQOL Group but QOL in the social relationships and environmental domains were comparable [27]. When comparing with another healthy sample in Hong Kong [20], our residents had slightly lower QOL in the physical health domain (score difference: - 0.48) but better QOL in the psychological (score difference: + 6.49), social relationships (score difference: + 2.52) and environmental domains (score difference: + 1.92). Our sample also showed better QOL scores in all domains compared with another Chinese population living in an urban community in China [26]. Using one SD below the mean as the cut-off standards for low QOL, however, a higher proportion in our study had poor QOL in all the four domains compared with this Chinese population [26].

Significant correlations between QOL in the four domains, overall QOL and general health were observed. Our findings are consistent with previous studies which showed that the four domains of QOL and perceived general health are interrelated. People with positive emotions or better QOL in the psychological domain evidence better physical health outcomes, such as fewer physical complaints, more exercise, longer sleeping hours and better sleep quality [28]. Increasing transient emotions can strengthen immune functioning and buffer the impact of stress which gives people better health [28, 29]. On the other hand, exposure to nature or green space has been found to improve people’s health and well-being by providing restoration from stress and mental fatigue [30]. In areas where 90% of the environment around the home was green, 5.3% less residents would feel unhealthy compared with areas in which only 10% of the environment was green [5]. Detrimental social relationships also play a role in physical and psychological health. An adverse family environment and lack of social support may result in depressive symptoms and subsequent psychological distress which in turn would affect one’s general health [31]. To facilitate social interactions and networking, a neighborhood with better built environment, such as street connectivity, traffic and pedestrian safety, improved air quality and greenery are necessary.

Studies have shown that people tend to have better mental health if they are living in an environment which is less affected by noise and increasing temperatures, with better air quality, plenty of vegetation and open spaces, adequate social and entertainment facilities, and safe to go out in the day and at night [31–33]. Our study found that there were significant differences in levels of satisfaction with air quality, noise pollution, parks and gardens, recreation and sports facilities, and promenade across the nine districts. The results also supported that residents who were moderately satisfied with the neighborhood environment had significantly higher QOL in the psychological domain compared with those who had low levels of satisfaction, however, the effect stopped at moderate satisfaction level. High levels of satisfaction with the neighborhood environment could not further raise QOL in the psychological domain.

The availability and accessibility of open spaces and leisure facilities, and the quality of air and noise condition could play an important role in the overall levels of satisfaction with the environment. Residents living in Districts 4 and 5 were moderately satisfied with the environment. Green and open spaces including a garden, a rest-area and a promenade are located in both districts. A relatively higher proportion of residents in District 4 were satisfied with the noise condition, and recreation & sports facilities while residents in District 5 were more satisfied with the promenade. Districts 8 and 9 are residential areas with lower SES but they are far from the busiest streets and traffic. The highest proportion of people satisfied with air quality, noise condition, recreation and sports facilities were found in District 8. This may explain why residents living in Districts 8 and 9 were highly and moderately satisfied with the environment, respectively. Districts 1, 3, 6 and 7 were the four districts with the lowest overall satisfaction with the environment. Main streets are running across Districts 1 and 6. Residents were most dissatisfied with air quality and noise pollution. Open spaces, parks and recreational facilities are obviously inadequate, or difficult to access in these districts. District 3 is close to the waterfront, nevertheless, residents were obviously dissatisfied with the air and noise qualities and the recreational and sports facilities nearby. Quality of green and open spaces are sometimes more crucial compared with quantity [34]. To understand the views of the residents in depth, interviews or focus group discussions are needed. The existing outdoor and recreational facilities may not be able to meet the needs of the residents, or they need to be renovated.

The sole effect of overall satisfaction with the neighborhood environment on QOL in the psychological domain can be explained by its potential influence on sleeping quality, green exercise, social contacts and cohesion. Green spaces help filtering pollution from the air and reduce air and ground temperature, which facilitate people to achieve a healthier duration of sleep. People do not sleep well have been observed to have adverse mood and cognitive performance. Moreover, increasing temperature has been associated with more aggressive behaviors and higher suicide rate, and stress-related disorders [35]. People who are living in a less crowded place or presence in a more natural environment would have more opportunities to involve activities in green places. Green exercise significantly decreases stressful events, loneliness and depression, while better QOL, happiness and social interaction are promoted [6, 11, 35]. A neighborhood with better built environment such as walkability and street connectivity can improve people’s mental health and well-being by increasing their social cohesion [29, 34, 36].

We found that potential health-related behaviors mediators including smoking status, alcohol drinking, physical activity levels, walking activity and total physical activity were significantly correlated with the overall satisfaction with the neighborhood environment. The quality of the neighborhood residential environment or neighborhood physical disorder has been found to be related to some of the health damaging behaviors such as cigarette smoking, alcohol and drugs [37, 38]. Neighborhood physical disorders are usually observed in areas with violence, poor safety at night, street littering, and traffic and neighborhood noise which could lead to psychological distress [39]. The high smoking rate in neighborhoods with physical disorders could be explained by the fact that there are fewer options for pleasurable activities, hence smoking is seen as one of the few pleasurable activities [38]. Other studies explained that disorderly and unsafe environments attract men whose SES is subordinate [40, 41]. The act of smoking can show a certain type of masculinity. In Hong Kong, however, due to land limitation and high housing price, most of the time mid-to high SES groups have to live in neighborhoods with undesirable environmental quality which they are not really satisfied with. More local studies are needed to investigate the associations of smoking, alcohol and environment, and compare with the western studies.

A systematic review showed that neighborhood environment had an association with walking for exercise while lack of equipment and facilities were only significantly related to sports and exercise activity [42]. These results are consistent with our findings which found that overall environmental satisfaction was significantly correlated with walking activity only, but not moderate and vigorous physical activities. Regarding walking activity, the supportiveness of neighborhood environmental attributes is crucial [36, 42]. People tend to walk more often if the air quality is less polluted, facilities are aesthetics, parks are safe, and street are well-connected. Moderate and vigorous physical activities are usually sports, endurance or strengthening exercises which require equipment and facilities. People can look for appropriate facilities out of their living districts, therefore, the influence of living environment is reduced.

In regard to the associations of health-related behaviors and QOL in the psychological domain, smoking status was the only health-related behaviors observed in this study. In previous studies, moderate alcohol drinking, however, was found positively associated with mental health because of the possibilities of social factors connected with alcohol [43]. Moderate-to-vigorous physical activity had also been reported to be positively correlated with psychological health-related QOL [44], but it has no relationship with QOL in the psychological domain in this study. Relationships between socio-demographic characteristics, such as age and sex, and QOL were also studied in previous studies, however, the findings were inconsistent. A study found that women aged 57–70 years exhibited significantly higher QOL in the physical, social relationships and environmental domains than men in the same age group, but nearly identical QOL in the psychological domain [45]. Another study found that women had significantly lower QOL in the psychological and social relationships domains possibly because women trended towards more depressed than men [46]. In future studies, relationships between socio-demographic factors, QOL and satisfaction with neighborhood environment can be considered.

The mediation analysis showed that the relationship of moderate satisfaction with the neighborhood environment and QOL in the psychological domain was partially mediated (16.41%) if the residents were non-smokers since the non-smoking status also significantly increased QOL in the psychological domain. QOL deteriorates with an increase in daily cigarette smoked [16, 47]. Non-smokers usually have higher QOL in the physical, mental and social functioning domains. Because of nicotine on neurotransmitter activity in the brain, smokers have been reported to have more depressive symptoms, as well as more complaints of weakness, headache, dizziness, pain and discomfort [16]. Smoking and depression are part of the same vicious cycle. Without any stress coping skills, they would smoke when they are stressed or depressed. The relief of the feelings of irritability and anxiety claimed by smokers could be simply the nicotine withdrawal effect after they have not smoked for a while. In many societies smoking is a discouraged or an unacceptable behavior causes some smokers to feel stressful and uneasy when smoking in public [48]. Same in Hong Kong, smoking is banned in many of the public indoor and outdoor areas. This combination of physical and mental influences could lower smokers’ QOL in the psychological domain. In other words, non-smokers usually have better QOL in the psychological domain. Other studies also identified that residential satisfaction and sense of community (i.e. the feeling that one is part of a larger dependable and stable structure) could mediate the impact of environment on psychological well-being, life satisfaction and positive affect [7, 49].

### Limitations

A convenience sampling method was used in this study and residents who participated were mainly those who stayed outdoors or used the facilities and amenities in the neighborhood. Those who spent most of their time indoors or at home were less likely to be approached. These limited our studying of the QOL of residents and their perspectives of the environment. A random sampling telephone interview could be an alternative in the future. The low specificity but high sensitivity of the IPAQ-SF and the potential of inaccuracy of self-reported data could also lead to the insignificant mediating effect of physical activity levels, walking activity and total physical activity observed on the relationship of satisfaction with neighborhood environment and QOL in the psychological domain. Individual in-depth interviews or focus group discussions together with questionnaire survey can help data triangulation to facilitate understanding of the reasons behind the ratings of the environment and open spaces. In future studies, objective data such as vegetation density, air pollutants and traffic noise should be considered to supplement the self-rated environmental data. Other factors like residential satisfaction, sense of community, and family and neighbor relationships which may potentially mediate the effect of neighborhood environment on QOL can be further investigated.

## Conclusions

This study assessed the QOL of residents living in the Kowloon peninsula of Hong Kong and the effect of overall satisfaction with the neighborhood environment on QOL. Our residents had lower QOL in the physical health and psychological domains but similar QOL in the social relationships and environmental domains compared to other countries. As the sample of residents were from different districts in Kowloon, their overall satisfaction with environment in the neighborhood varied. A significant relationship between satisfaction with the neighborhood environment and QOL in the psychological domain was found. The non-smoking status of the residents partially mediated the effect of moderate environmental satisfaction on the psychological domain. In a dense city like Hong Kong where green and open spaces are limited, refrain from smoking seems to be able to lower the influence of neighborhood environment on people’s QOL in the psychological domain to a certain extent. Health promotion and smoking cessation programs can stress the importance of not smoking in enhancing QOL in the psychological domain. This study also provides policy makers and health administrators with evidence-based information on how physical and built environment can influence residents’ QOL especially in the psychological domain as well as facilitating the development of environmental interventions and policy recommendations.

## Abbreviations

IPAQ-SF: International Physical Activity Questionnaire- Short Form
MET: Metabolic Equivalent of Task
QOL: Quality of life
SES: Social Economic Status
WHOQOL-BREF: World Health Organization Quality of Life: Brief Version
